# Supplementary material for: A Dilp8-dependent time window ensures tissue size adjustment in Drosophila
Source: Nat Commun. 2022 Sep 26;13:5629. doi: 10.1038/s41467-022-33387-6 (PMC9512784; doi:10.1038/s41467-022-33387-6)
Supplement: Supplementary file 1 — Supplementary Information [file 41467_2022_33387_MOESM1_ESM.pdf]

SUPPLEMENTARY INFORMATION

Supplementary Figure 1

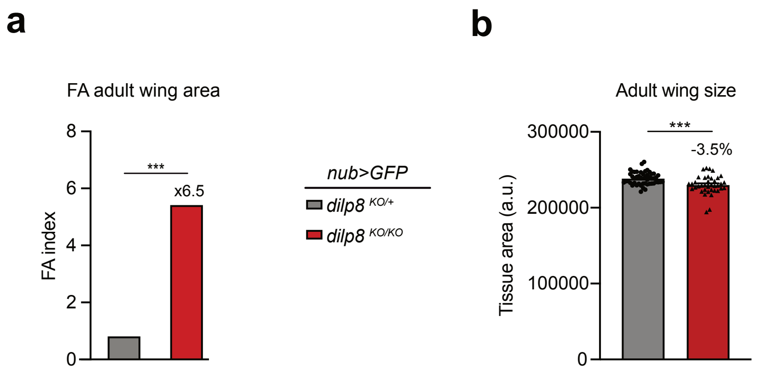

(a) FA indexes in adults of the same genotypes and issued from the same crosses as presented in Figure 1. n=24 independent pairs of wings for *dilp8*$^{KO/+}$*, nub>GFP* controls and n=19 for *dilp8*$^{KO/KO}$*, nub>GFP* animals. ***p=0.0001, two-tailed F-test. (b) Average adult wing area for the same animals as (a). Error bars represent SEM. ***p = 0.0006, two-tailed t-tests. Source data are provided as a Source Data file.

Supplementary Figure 2

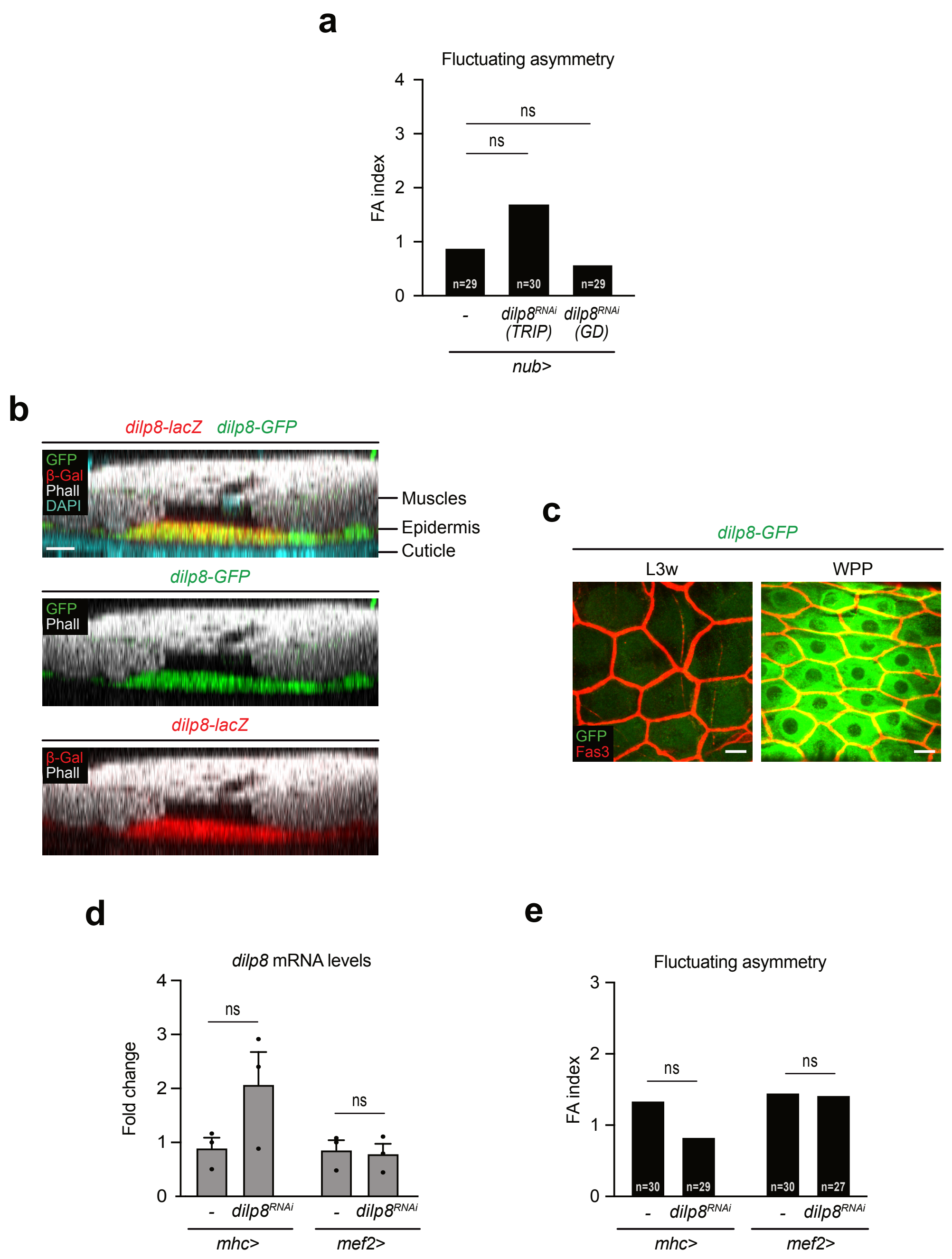

(a) FA indexes of adult wings upon RNAi-mediated downregulation of *dilp8* in the wing imaginal discs using *nub-GAL4*. n values indicate the number of independent wing pairs analyzed; ns=not significant, two-tailed Levene's test. (b) Maximal projection lateral view of a fillet preparation at the WPP stage showing expression of the *dilp8-lacZ* (in red) and *dilp8-GFP* (in green) transcriptional reporters in the epidermis. Phalloidin (Phall) staining marks the muscles and DAPI is used here to stain the cuticle. Scale bar represents 20 microns. The experiment was repeated independently more than 3 times with similar results. Brightness and contrast adjustments were performed without altering signal localization. (c) Maximal projections of fillet preparations showing expression of the *dilp8-GFP* reporter at the wandering L3 stage (L3w) and at the WPP stage. Fasciclin 3 (Fas 3, in red) marks the outer membranes of epidermal cells. Scale bars represent 20 microns. This experiment was repeated independently 2 times with similar results. Brightness and contrast adjustments were performed equally on control and experimental conditions and without altering signal localization. (d) Measurement of *dilp8* mRNA levels by qRT-PCR on whole animals at the WPP stage upon RNAi-mediated downregulation of *dilp8* (*UAS-dilp8^{RNAi}* TRIP line) in the muscles. Values are expressed as fold changes relative to controls without RNAi. Error bars represent SEM. ns=not significant, two-tailed t-tests. n = 3 biologically independent samples were analyzed for all genotypes. (e) FA indexes of adult wings upon RNAi-mediated downregulation of *dilp8* (UAS-*dilp8^{RNAi}* TRIP line) in the muscles. n values indicate the number of independent wing pairs analyzed; ns=not significant, two-tailed F-tests. Experiments in (a, d, e) were done at 29°C. Source data are provided as a Source Data file.

Supplementary Figure 3

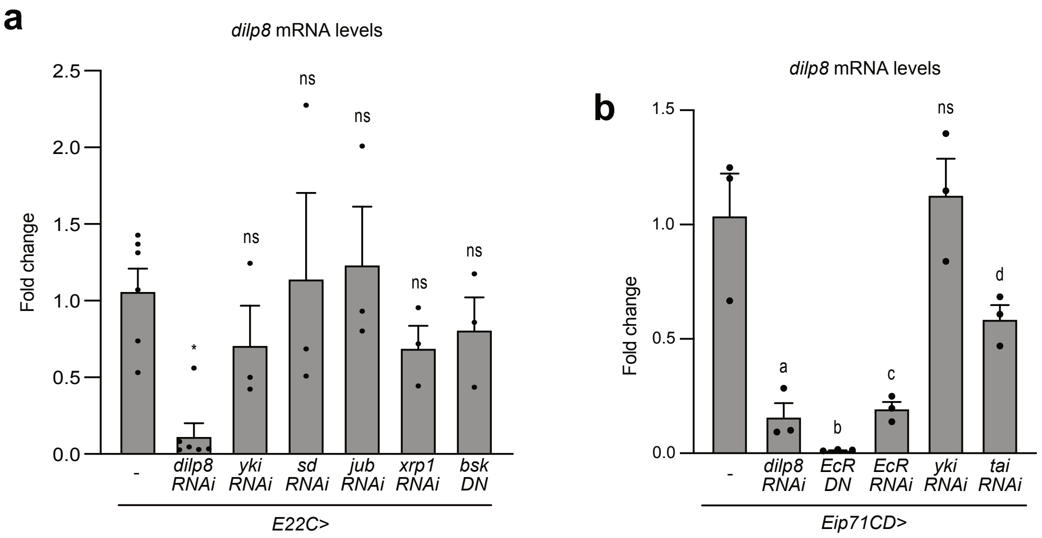

(a) and (b) Measurement of *dilp8* mRNA levels by qRT-PCR on whole animals of the indicated genotypes at the WPP stage. Values are expressed as fold changes relative to controls without RNAi. Error bars represent SEM. *p=0.0025 , a: p<0.0001, b: p<0.0001, c: p=0.0001, d: p=0.0119 and ns=not significant, one-way ANOVA. Experiments were done at 25°C. n = 6 biologically independent samples were analyzed for the *E22C>* control and *E22C>dilp8*^{RNAi} genotypes and n=3 for all the other genotypes. Source data are provided as a Source Data file.

Supplementary Figure 4

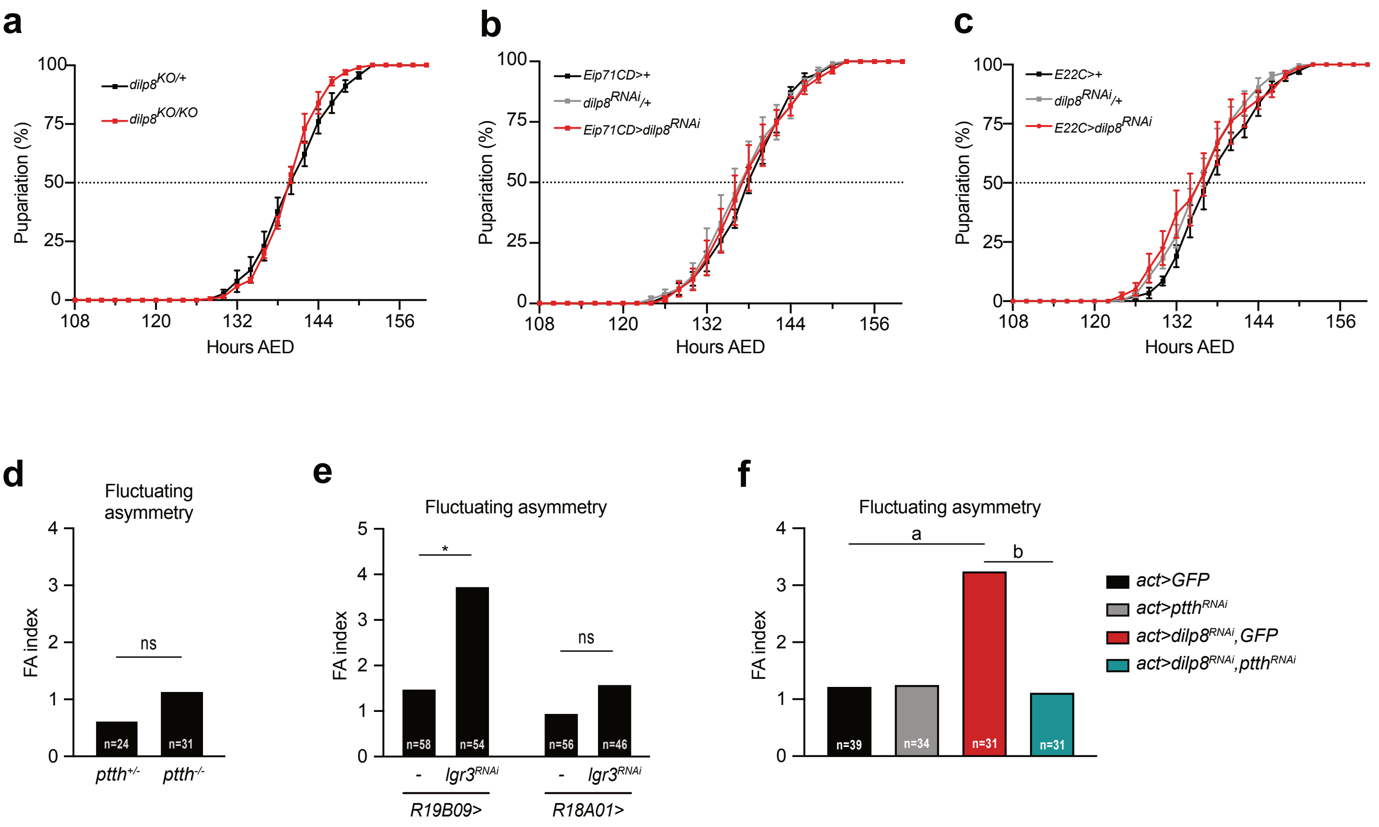

(a-c) Pupariation curves of populations of the indicated genotypes. n = 5 biologically independent experiments (with 30 to 50 animals each) were analyzed for all genotypes. Error bars represent SEM. AED: after egg deposition. (d) FA indexes of adult wings in control heterozygous and homozygous mutant animals for *ptth*. n values indicate the number of independent wing pairs analyzed. ns = not significant, two-tailed F-test. (e) FA indexes of adult wings upon RNAi-mediated downregulation of the Dilp8 receptor Lgr3 (*UAS-lgr3^{RNAi}* line) in two different sets of Lgr3-positive neurons (expressing the *R19B09-GAL4* and the *R18A01-GAL4* drivers, respectively). n values indicate the number of independent wing pairs analyzed. *p = 0.0155 and ns = not significant, Levene's tests. (f) FA indexes of adult wings of the indicated genotypes, showing that *dilp8* loss-of-function can be rescued by decreasing systemic ecdysone levels through ubiquitous downregulation of *ptth*. n values indicate the number of independent wing pairs analyzed. a: p = 0.0084, b: p = 0.0026, two-tailed F-tests. Source data are provided as a Source Data file.

Supplementary Table 1

Target	Sense primer	Antisense primer
rp49	5’-CTTCATCCGCCACCAGTC-3’	5’-CGACGCACTCTGTTGTCG-3’
dilp8	5’-CGACAGAAGGTCCATCGAGT-3’	5’-GATGCTTGTTGTGCGTTTTG-3’
EcR	5’-ACCAGCGTTTACAAAGATACCC-3’	5’-CATCATCACCTCCGACGAG-3’
E78	5’-CATGTGGCCCGGTTGATC-3’	5’-CGTTGACAAAGTCAGAATCGTAGAG-3’
E74A	5’-TGAGACGCGAGGAATACCCTGGAC-3’	5’-AACTGCCAGCGTGTAGCCGTTTCC-3’
E75A	5’-TTACGGCGTGCATTCCT-3’	5’-GGGCGATACTGGATCTTTTG-3’
E74B	5’-GCGTGCGTCCGTGTAAGTG-3’	5’-CCCAATACCAGTGCACCAGTT-3’
DHR3	5’-AATATTGTAGACTGCAAAAGTGCCTA-3’	5’-GGACATCCTGCCGAACTTTA-3’
DHR4	5’-TGCTCTCCCACATACCAGAGA-3’	5’-CACGAAGGGCACATAGAACA-3’
ftz-f1	5’-TTGCCGCTTTTTAAGAACATTT-3’	5’-TGACATTTTAATCTCTCCAGGAGTATC-3’
br	5’-TCTGTGACTCGGTGACATTTGCGA-3’	5’-TTACTAGACCGCTTGCCGGATTGT-3’
