# Supplementary material for: A Dilp8-dependent time window ensures tissue size adjustment in Drosophila
Source: Nat Commun. 2022 Sep 26;13:5629. doi: 10.1038/s41467-022-33387-6 (PMC9512784; doi:10.1038/s41467-022-33387-6)
Supplement: Supplementary file 3 — Description of Additional Supplementary Files [file 41467_2022_33387_MOESM3_ESM.pdf]

Description of Additional Supplementary Files

File name: Supplementary Data 1

Description: Instructions and demo for code associated with this paper.
