# Supplementary material for: A Dilp8-dependent time window ensures tissue size adjustment in Drosophila
Source: Nat Commun. 2022 Sep 26;13:5629. doi: 10.1038/s41467-022-33387-6 (PMC9512784; doi:10.1038/s41467-022-33387-6)
Supplement: Supplementary file 4 — Supplementary Data 1 [file 41467_2022_33387_MOESM4_ESM.zip › Supplementary Software Information/Instructions_AutomatedWingSegmentation.pdf]

Automated segmentation of *Drosophila* wings

1. Area measurement and Fluctuating asymmetry quantification tool (FAQT) 1

1.1. General description 1

1.2. Instructions for use: Area Measurement 2

1.3. Instructions for use: FAQT 5

2. Wing segmentation visualization and correction tools 8

2.1. General description 8

2.2. System and software requirements 9

2.3. Instructions for use 10

1. Area measurement and Fluctuating asymmetry quantification tool (FAQT)

1.1. General description

We provide two codes for measuring wing area or fluctuating asymmetry. The first one, AreaMeasurement, contains all files required to perform a UNET automated segmentation of *Drosophila* wings and outputs an Excel file with the pixel area values for each wing. The second one, FAQT (Fluctuating Asymmetry Quantification Tool), contains all files required to perform a similar UNET automated segmentation of *Drosophila* wing pairs and outputs an Excel file with the pixel area values for each wing, as well as asymmetry values for each pair.

These Google Colab notebooks automatically segment the adult *Drosophila* wing area, based on the associated deep-learning trained model "DeepWingSegmentationModelUNET" (details provided in the "Methods" section of the article). Importantly, segmentation works on images taken with specific settings (see next paragraph).

Results can be visualized, and errors can be corrected using the associated correction tools generated for this purpose (see hereafter in section 2). As an example of the full process, see the DEMO file.

System requirements

The codes are executed in a Google Colab environment; therefore, the only requirement is a Google account. Files must be copied/transferred to Google Drive (see instructions), and

double click on the code icon automatically opens Google Colab. It is recommended to have a Google Colab Pro account, with GPU and high RAM access. This greatly shortens the time for the code to execute, compared to a regular Google account.

Image requirements

Raw images of the wings were acquired as TIF files with a 1024 x 768 resolution using a MZ16-FA Leica Fluorescence Stereomicroscope with a DFC-490 Leica digital camera (Bright-field mode, 50% illumination intensity, 10.5 exposure, 2.3 gain, 152 saturation and 1.20 gamma). Respecting these settings and resolution was key in our hands for this code to perform a proper segmentation of the *Drosophila* wing. With another equipment, tests will be needed to find the appropriate settings.

The presence of bubbles or hairs close to the wings might induce mistakes in the segmentation process which can be manually corrected afterwards.

1.2. Instructions for use: Area Measurement

1) Download the "Area_Measurement" folder from the Zenodo repository:
Varun Kapoor. (2022). Drosophila Wing Area Measurement and Fluctuating Asymmetry Quantification Tool (FAQT) (Version 2). Zenodo.
https://doi.org/10.5281/zenodo.7026011

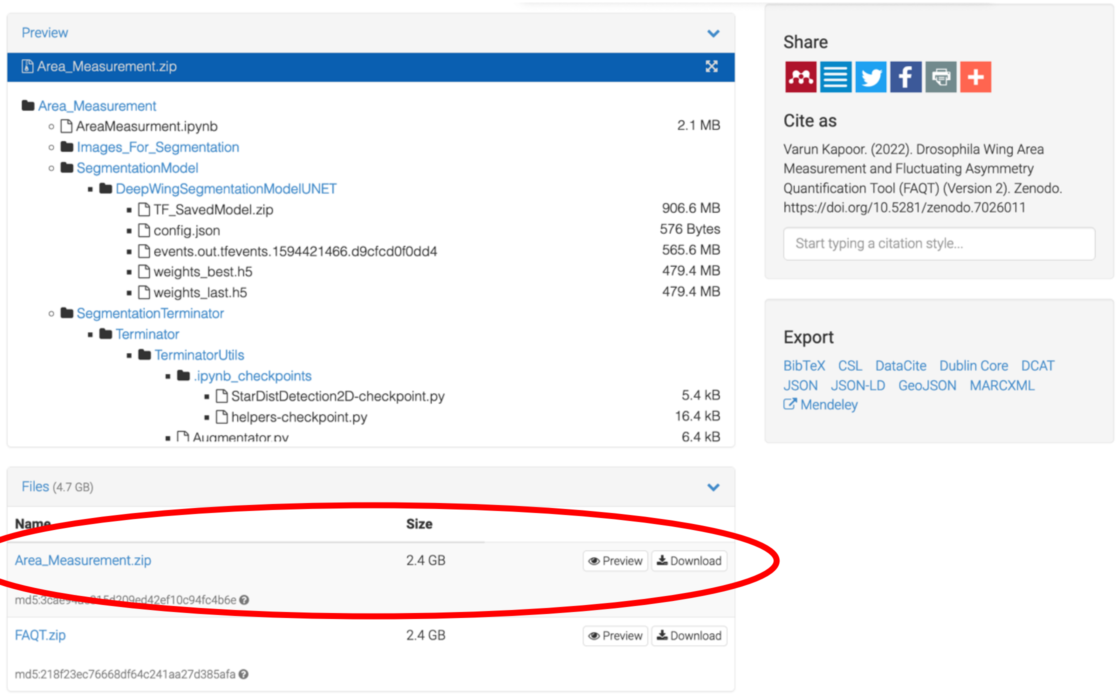

2) Upload the "Area_Measurement" file to your Google Drive and open the folder.

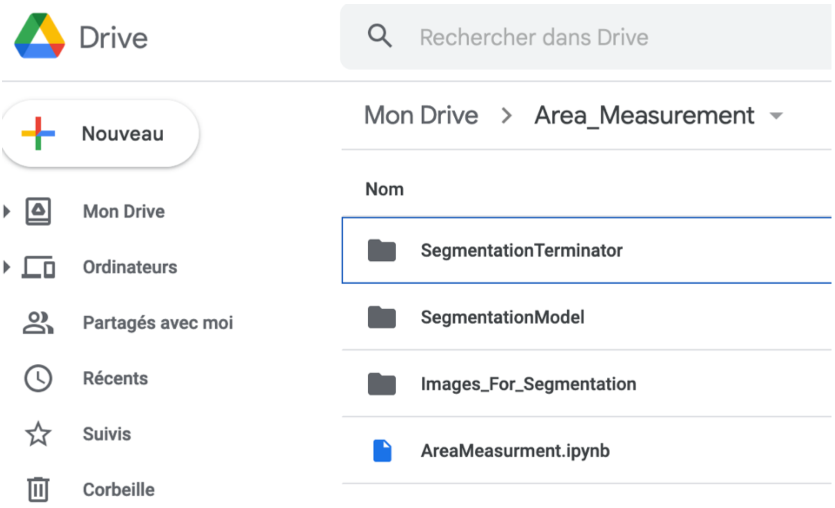

3) Upload wing images to be processed to the “Images_For_Segmentation” folder.

4) Open the AreaMeasurment.ipynb code from the Area_Measurement folder using the Google Colab platform (a simple double click will open it directly with Colab).

5) Run the program by clicking on Runtime>Run all (or Restart and run all). The first cell will ask for your permission to connect to your Google Drive account in order to access the files directories. Click on “Connect to Google Drive”, choose your account and “Allow”.

6) The code will run.

7) After the code has run, results folder will be saved in the Images_For_Segmentation directory. There, you will find the subfolders AreaResults (Area.csv file with quantitative information which can directly be opened with Excel) and MaskResults (binary segmentation mask for each wing image).

8) The name of the source folder (or Masterdir, “Images_For_Segmentation”) can be easily modified in the code where indicated (see screenshot). If needed, modify the name of the source folder in the first command line of that cell.

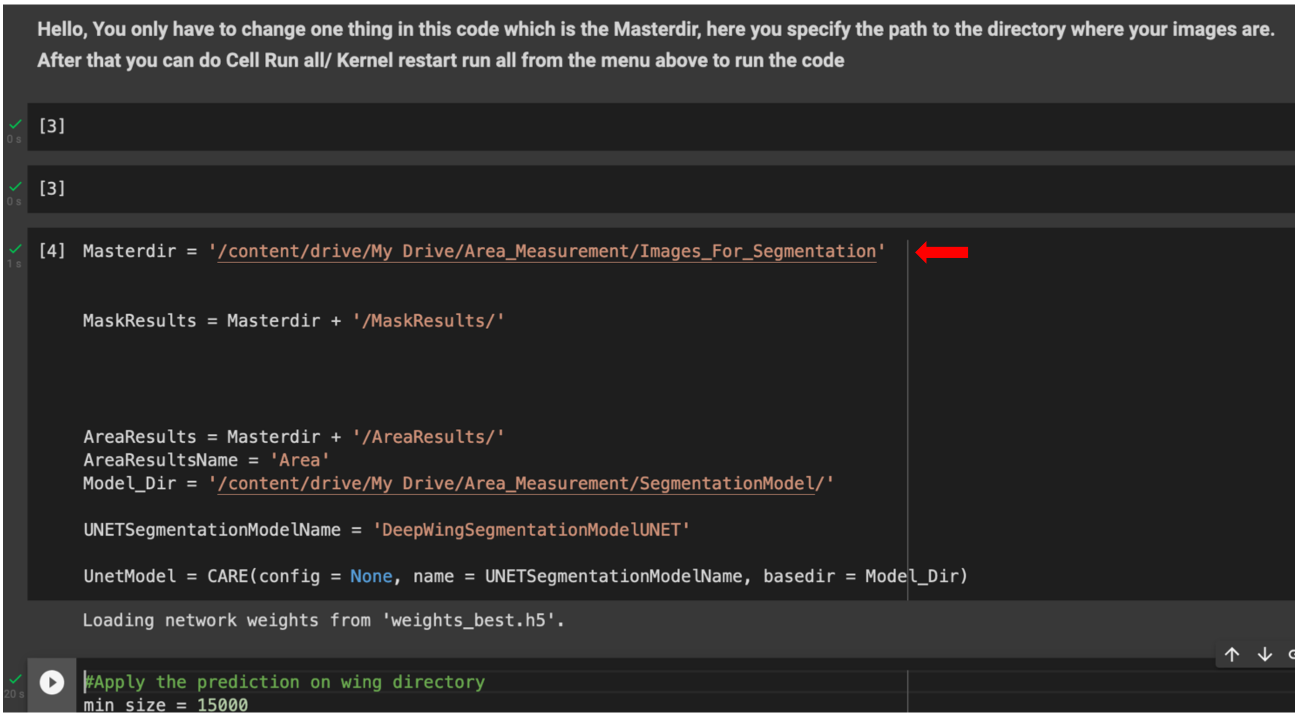

9) At the end of the code, the newly created masks are shown, together with the values calculated for wing area.

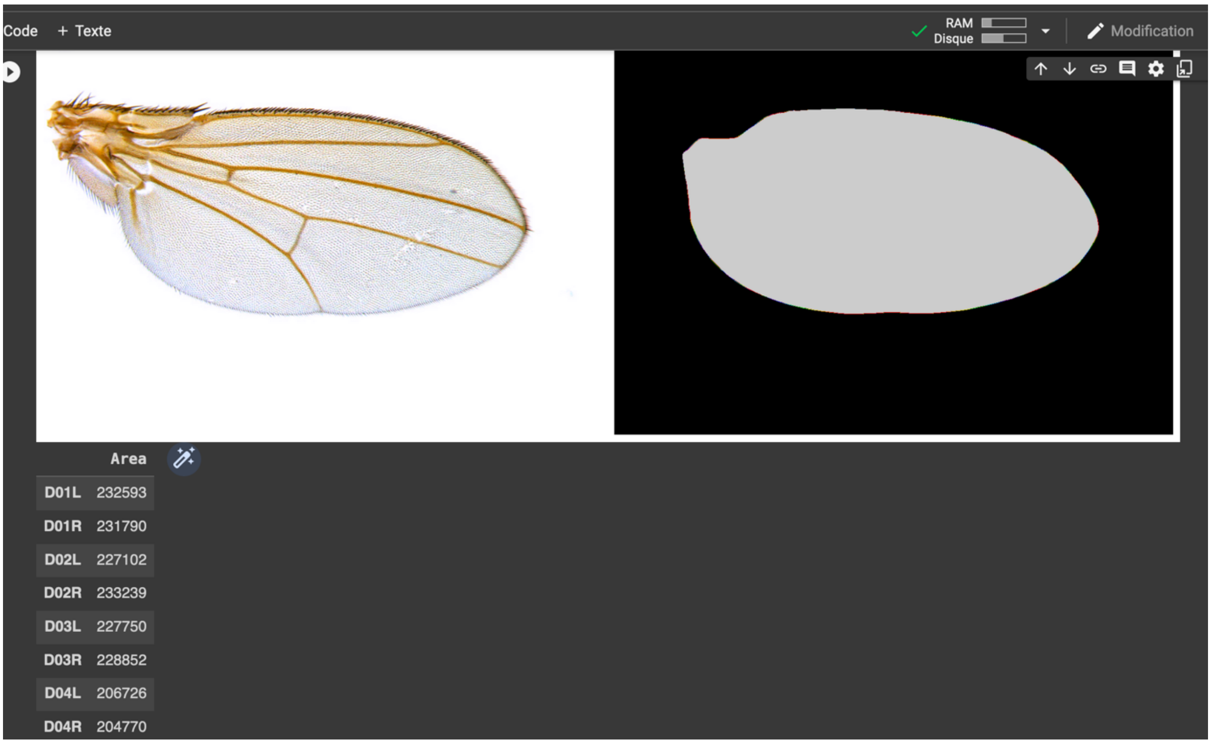

10) Finally, download the folder with images and results ("Images_For_Segmentation" containing pictures, AreaResults and MaskResults folders) to a given location on your computer to proceed with the visualization and correction tool.

1.3. Instructions for use: FAQT

1) Download the “FAQT” folder from the Zenodo repository:
Varun Kapoor. (2022). Drosophila Wing Area Measurement and Fluctuating Asymmetry Quantification Tool (FAQT) (Version 2). Zenodo.
https://doi.org/10.5281/zenodo.7026011

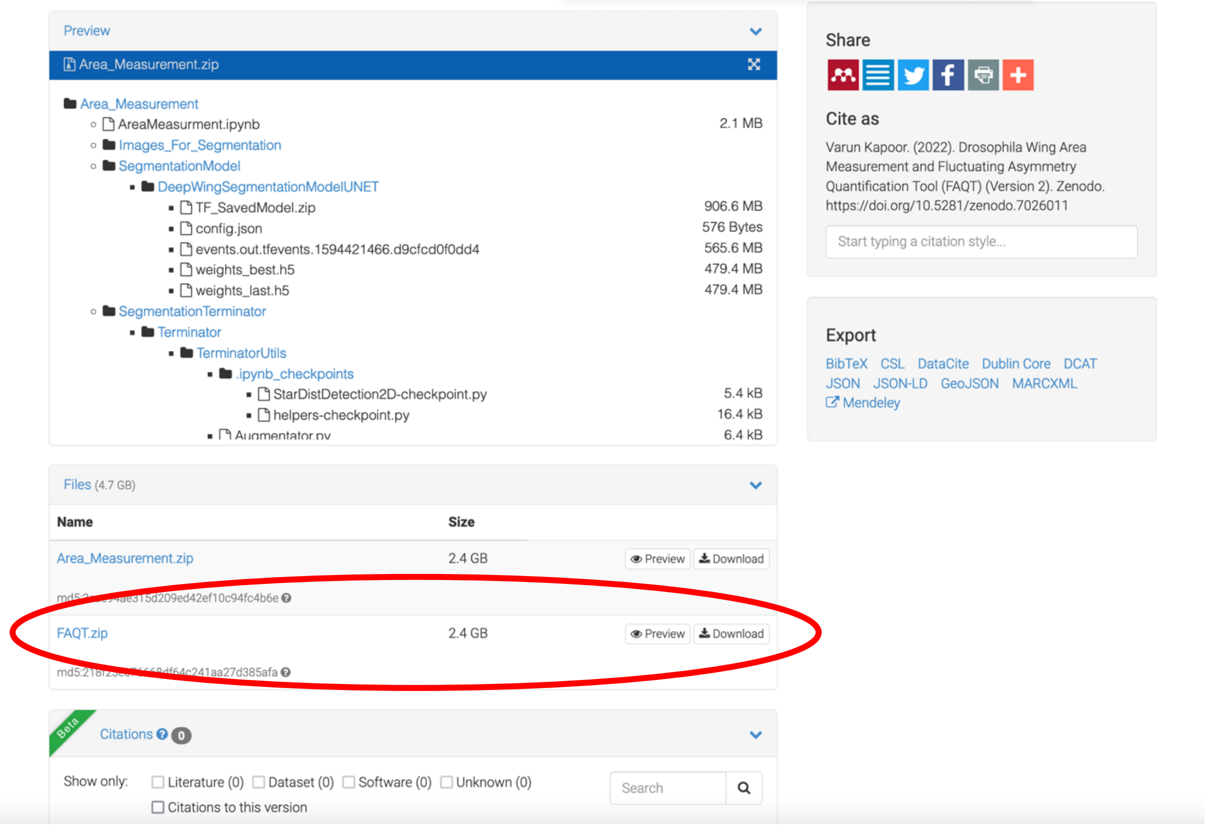

2) Upload the “FAQT” file to your Google Drive.

3) Upload wing images to be processed to the “Images_For_FAQT” folder. **Important note**: Right- and Left-wing images name must end with “R” (right) and “L” (left) respectively so that the program can identify pairs. Thus, file names will have the following format: condition_pairnumberR. Example: dilp8KO_01L and dilp8KO_01R for the two wings of a given pair.

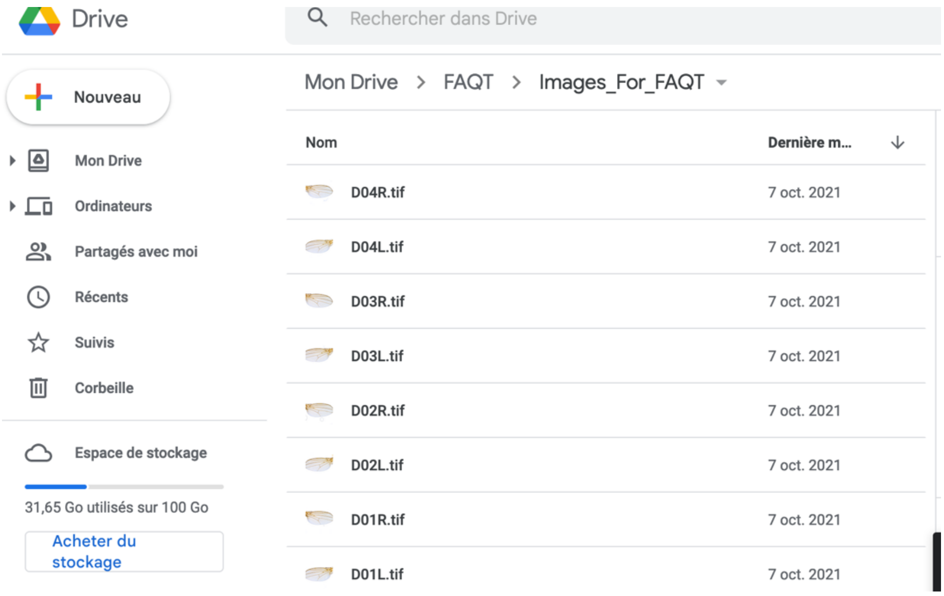

4) Open the ColabFAQT.ipynb code from the FAQT folder using the Google Colab platform (a simple double click will open it directly with Colab).

5) Run the program by clicking on Runtime>Run all (or Restart and run all). The first cell will ask for your permission to connect to your Google Drive account in order to access the files directories. Click on "Connect to Google Drive", choose your account and "Allow".

6) The code should run. However, sometimes the following error occurs mentioning that the version of 'keras' is too old.

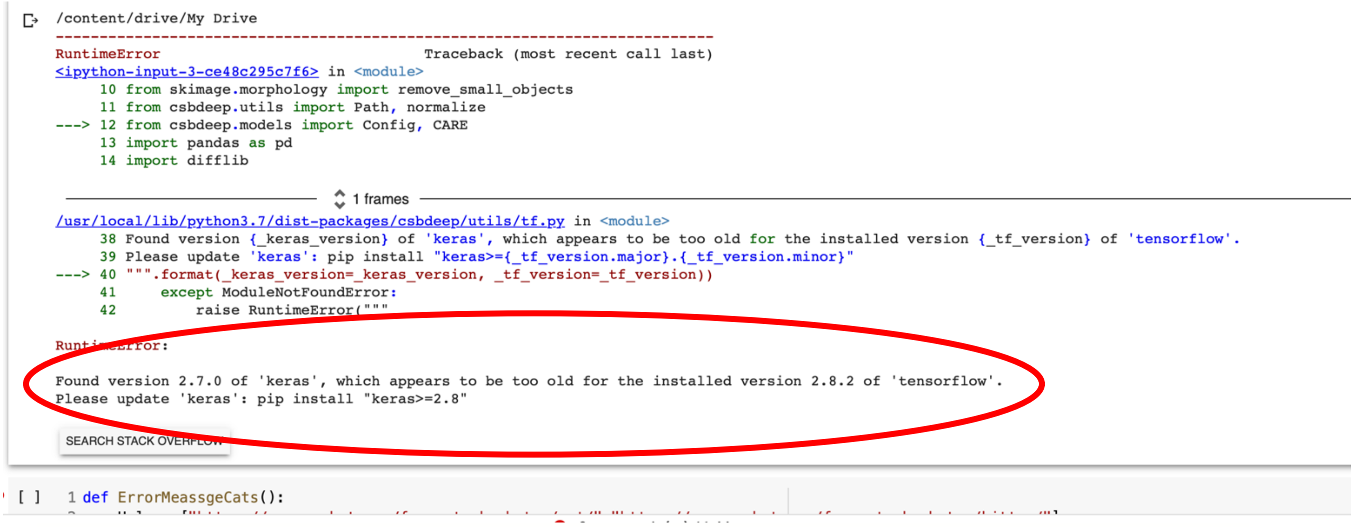

If this happens, you must update the version of keras in the code as requested. In the previous screenshot for example, you are asked to replace 2.7 by 2.8. This line is the 3rd one of the cell number 2 (`!pip install keras==2.8`):

+ Code + Texte

```
[1]
from google.colab import drive
drive.mount('/content/drive', force_remount = True)
%tensorflow_version 2.x
```

```
Mounted at /content/drive
Colab only includes TensorFlow 2.x; %tensorflow_version has no effect.
```

Double-cliquez (ou appuyez sur Entrée) pour modifier

```
!pip install tiffile
!pip install csbdeep
!pip install keras==2.8
!pip install cdifflib
!pip install scikit-image
```

7) After the code has run, results will be saved in the “Images_For_FAQT” folder in Google Drive as a new folder “For_Napari_Correction”. There, you will find the subfolders AsymmetryResults (Asymmetry.csv file with quantitative information) and MaskResults (binary segmentation masks for each wing image). In the Asymmetry.csv file the following parameters are available: "RightArea", "LeftArea", "Right-Left", "Asymmetry" (right wing area, left wing area, difference between the right- and left-wing areas, and percentage area difference between left and wing areas normalized to the left and right area mean, respectively). In addition, the code has generated new versions of the wing pictures, where orientation has been corrected and all wings have been flipped in the same direction to prevent any directional bias.

The names of the source folder (or Masterdir, “Images_For_FAQT”) and the new one created automatically to save the results (or Savedir, “For_Napari_Correction”; and subfolders) can be easily modified in the code in the 5th cell. If needed, modify the name of the source folder in the first command line of that cell, and the name of the results folder in the 2nd line. However, keeping this nomenclature will be helpful to use the visualization and correction tool more easily.

```
1 Masterdir = '/content/drive/My Drive/FAQT/Images_For_FAQT/'
2 Savedir = '/content/drive/My Drive/FAQT/Images_For_FAQT/For_Napari_Correction/'
3 Model_Dir = '/content/drive/My Drive/FAQT/SegmentationModel/'
4 UNETSegmentationModelName = 'DeepWingSegmentationModelUNET'
5
6 UnetModel = CARE(config = None, name = UNETSegmentationModelName, basedir = Model_Dir)
7 Path(Savedir).mkdir(exist_ok = True)
8
9 LeftName = 'L'
10 RightName = 'R'
11 file_extension = '*.tif' #change extension here if files have other extension
12 show_after = 25  #visualization after this number of images
13 min_size = 15000
14 MaskResults = Savedir + '/MaskResults/'
15 AsymmetryResults = Savedir + '/AsymmetryResults/'
16 AsymmetryResultsName = 'Asymmetry'
17 Path(MaskResults).mkdir(exist_ok = True)
18 Path(AsymmetryResults).mkdir(exist_ok = True)
```

8) At the end of the code, a small recap is displayed with a plot of the values obtained for asymmetry. It allows to confirm that the pairs of masks have been correctly assigned.

```
MaskD01L MaskD01R
MaskD02L MaskD02R
MaskD03L MaskD03R
MaskD04L MaskD04R
Positive Count 2
Negative Count 2
```

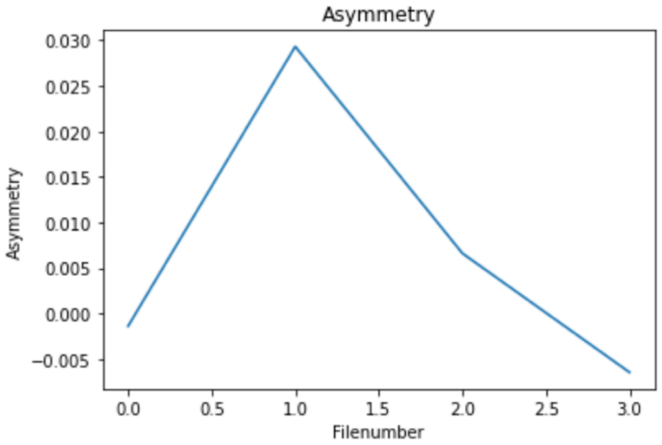

9) Finally, download the folder with the results ("For_Napari_Correction") to a given location on your computer to proceed with the visualization and correction tool.

2. Wing segmentation visualization and correction tools

2.1. General description

These are two Napari-based visualization and correction tools for *Drosophila* wing images segmented using the codes presented in 1. In this correction tools, pictures and masks are loaded and correction is performed using the paintbrush tool of Napari. All the corrections are automatically saved (masks and area/asymmetry results) after the user exits the viewer. These tools are executed locally on a computer (Jupyter notebooks).

We have included a code for plain wing area correction (AreaCorrectionTool.ipynb) and another for Wing Area correction of paired left and right wings (AsymmetryCorrectionTool.ipynb) obtained with FAQT.

In the AreaCorrectionTool, the segmentation masks in the MaskResults subfolder and the Areas.csv file in the AreaResults subfolder will be over-written with the corrected binary masks and area values, respectively. In the AsymmetryCorrectionTool, the segmentation masks in the MaskResults subfolder and the Asymmetry.cvs file in the AsymmetryResults subfolder will be over-written with the corrected binary masks and area and asymmetry values, respectively.

2.2. System and software requirements

We have used these tools in three different types of computers:

- Very recent MacBook Pro laptops (2021) running with macOS Monterey
- Old iMac (2010) running with macOS El Capitan
- PC running with Windows 11

Different versions of the Anaconda software and packages were used depending on the computer's configuration (see tables). For the recent mac laptops, the latest version of the Anaconda navigator (2.2.0) and default packages versions it contains were used. For the old iMac and PC, Anaconda Navigator 2020.02 (https://repo.anaconda.com/archive/) and older packages were installed.

1) Download the Anaconda Navigator (new version: https://www.anaconda.com/products/distribution; old version 2020.02: https://repo.anaconda.com/archive/) and follow the installing instructions.

2) Install Napari package by following the instructions in https://napari.org/stable/tutorials/fundamentals/installation.html.

3) Only if using the older version of Anaconda: go to the Environments tab and install the following packages:

Package	Version
ipython	7.23.1
Napari	0.4.3
Tifffile	2020.6.3
Pandas	1.0.1
Numpy	1.18.1
ipykernel	5.1.4
Tornado	6.0.3

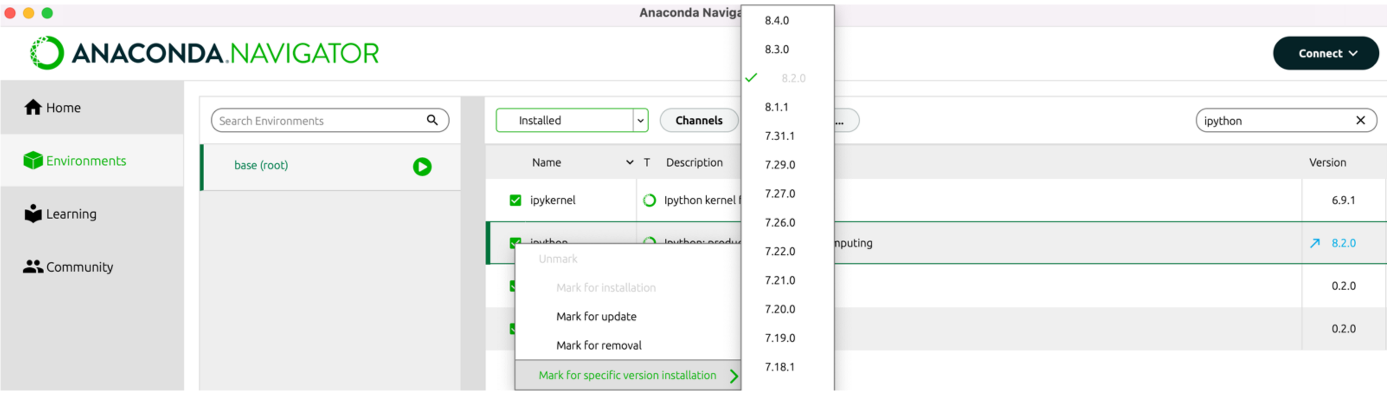

2.3. Instructions for use

1) Download the AsymmetryCorrectionTool.ipynb source code from the Zenodo repository:

Varun Kapoor. (2022). Drosophila wing segmentation visualisation and correction tools (Version 4). Zenodo. https://doi.org/10.5281/zenodo.7025439

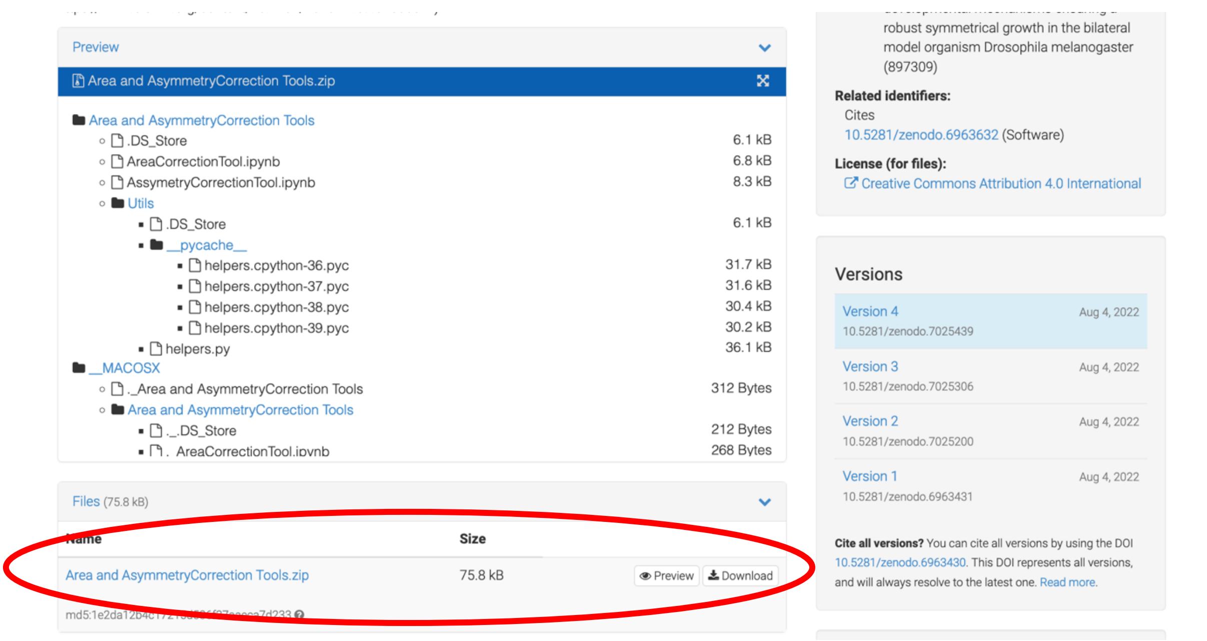

2) Go to the Home tab of the Anaconda Navigator and launch Jupyter Notebook.

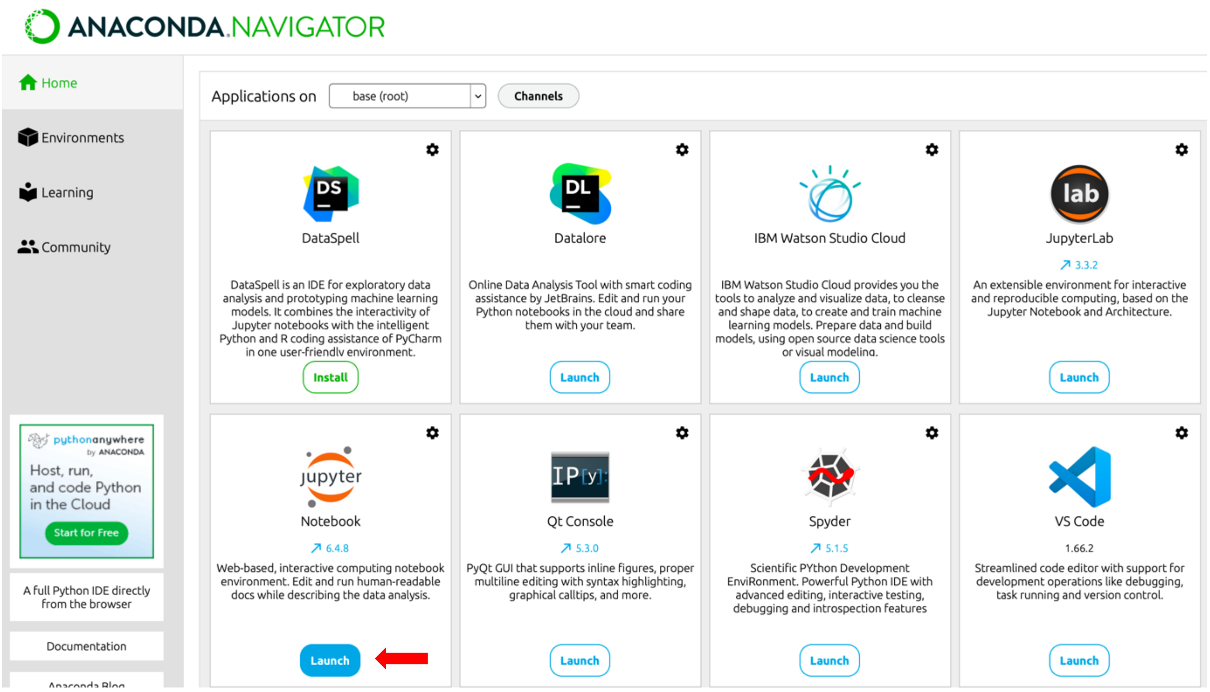

3) Locate the folder and open the correction tool needed (AreaCorrectionTool.ipynb if segmentation was done with AreaMeasurement; AssymetryCorrectionTool.ipynb if segmentation was done with FAQT).

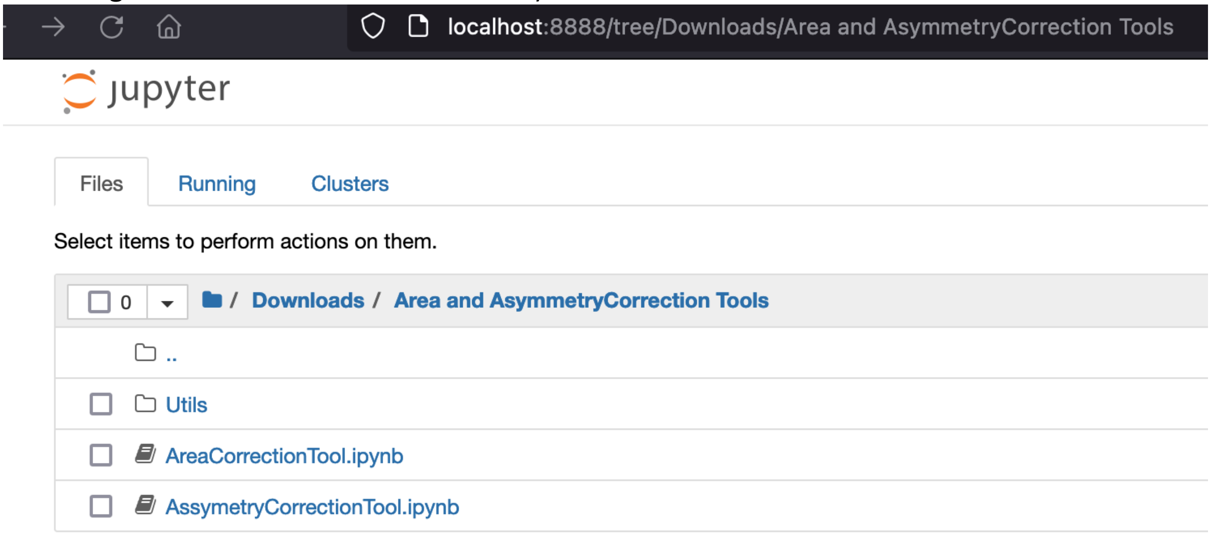

4) In the 2nd cell of the code, the **Masterdir** code line, specify the folder directory containing the results of the segmentation process (the "Images_For_Segmentation" folder downloaded after using AreaMeasurement; or the "For_Napari_Correction" folder downloaded after using FAQT).
For example:

Entrée [2]:
```
Masterdir = '/Users/LauraBoulan/Downloads/For_Napari_Correction/'


LeftName = 'L'
RightName = 'R'

MaskResults = Masterdir + '/MaskResults/'

AsymmetryResults = Masterdir + '/AsymmetryResults/'
AsymmetryResultsName = 'Asymmetry'
OverlayResults = MaskResults + '/Overlays/'
```

5) Execute the code by clicking Kernel>Restart & Run all. A Napari window containing the pictures+Masks overlays will open.

6) Visualize the masks and correct potential segmentation errors using the erase, paint or fill tools.

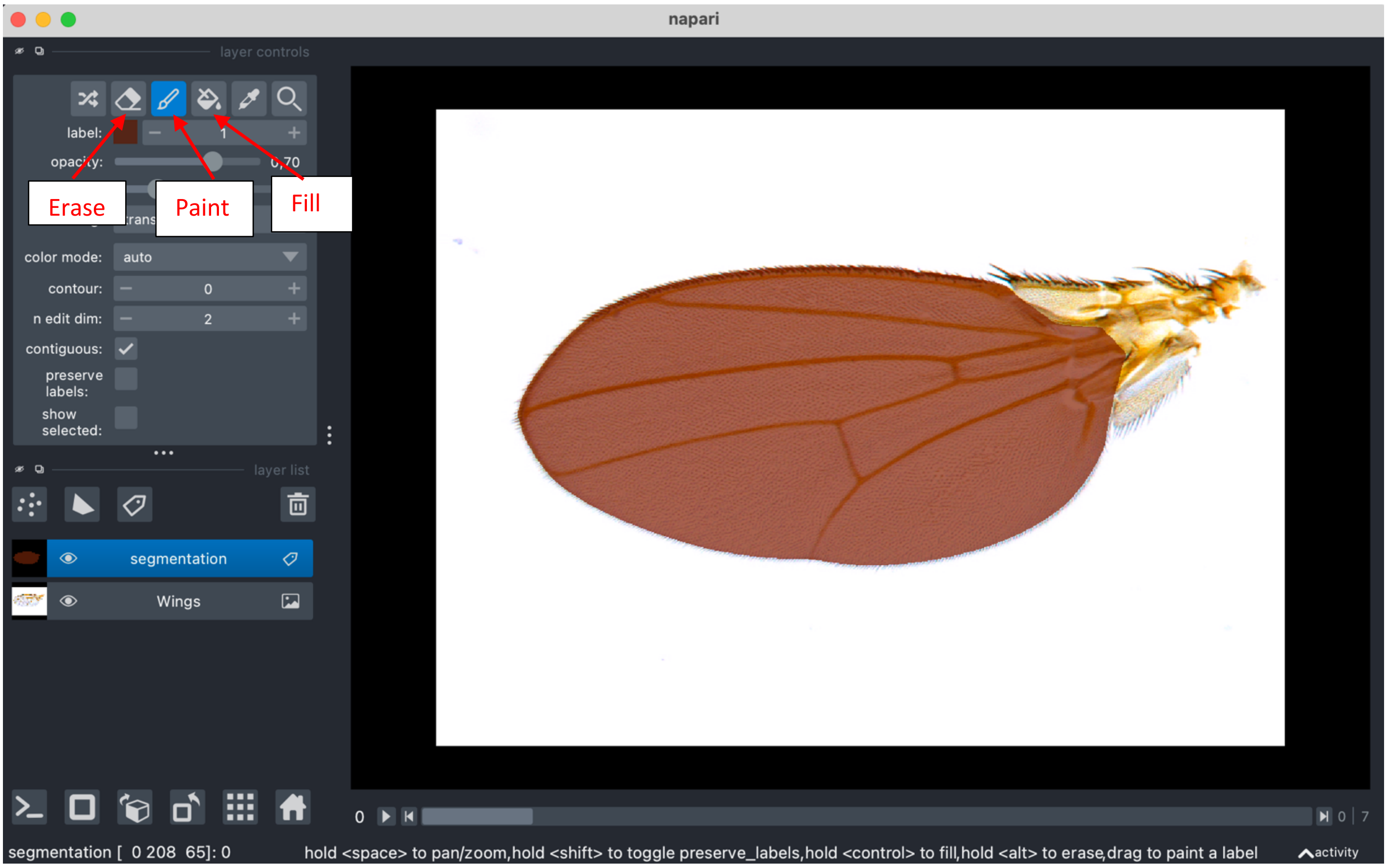

7) Close the Napari window, thereby saving the results. The segmentation masks and the wing area or asymmetry measurements will be replaced with the corrected data.
