# Supplementary material for: A Dilp8-dependent time window ensures tissue size adjustment in Drosophila
Source: Nat Commun. 2022 Sep 26;13:5629. doi: 10.1038/s41467-022-33387-6 (PMC9512784; doi:10.1038/s41467-022-33387-6)
Supplement: Supplementary file 4 — Supplementary Data 1 [file 41467_2022_33387_MOESM4_ESM.zip › Supplementary Software Information/Demo/Demo.pdf]

Here is an example of the segmentation process using the FAQT code to measure wing FA. There are 4 pairs of wings of the “D” genotype named D01L, D01R, etc…

Following the instructions file, copy the wings picture to the “Images_For_FAQT” folder on Google Drive and run the ColabFAQT code.

Run time was 2 minutes for 4 pairs of wings with the free version of Google Colab. Running time increases with the number of wings, but using the Pro version of Colab reduces the time to few minutes even with >60 wing pairs.

The result displayed at the end of the code is the following:

```
MaskD01L MaskD01R
MaskD02L MaskD02R
MaskD03L MaskD03R
MaskD04L MaskD04R
Positive Count 2
Negative Count 2
```

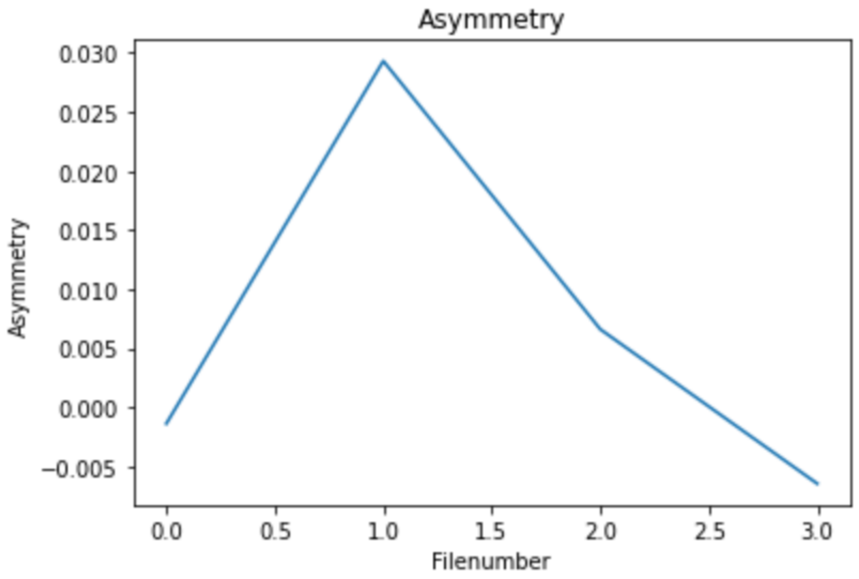

After downloading the results folder called “For_Napari_Correction”, the results are displayed in the Asymmetry.csv file which can be opened with Excel (use Data>Convert to split the columns). Before correction, the results are the following:

	RightArea	LeftArea	Right-Left	Right+Left	Assymmetery
MaskD01L	231933	232251	-318	464184	-0.0013701463212863864
MaskD04L	205017	206343	-1326	411360	-0.006446907817969662
MaskD02L	229806	227599	2207	457405	0.009650091275784043
MaskD03L	228996	227486	1510	456482	0.006615813986093647

To visualize the segmentation results and correct the masks if needed, let’s run the Visualization and Correction tool as explained in the Instructions file. Because the aim is to measure FA, I choose to run the AssymetryCorrectionTool.ipynb jupyter notebook. You just have to indicate where the Masterdir folder is located. For example:

Entrée [2]:

```
Masterdir = '/Users/LauraBoulan/Downloads/For_Napari_Correction/'


LeftName = 'L'
RightName = 'R'

MaskResults = Masterdir + '/MaskResults/'

AsymmetryResults = Masterdir + '/AsymmetryResults/'
AsymmetryResultsName = 'Asymmetry'
OverlayResults = MaskResults + '/Overlays/'
```

Then click on Kernel>Restart and run all, and the Napari window opens with the overlays (pictures+masks). Check all the wings and correct for possible errors (bubbles included in the mask, etc…). The overlays should be as shown here:

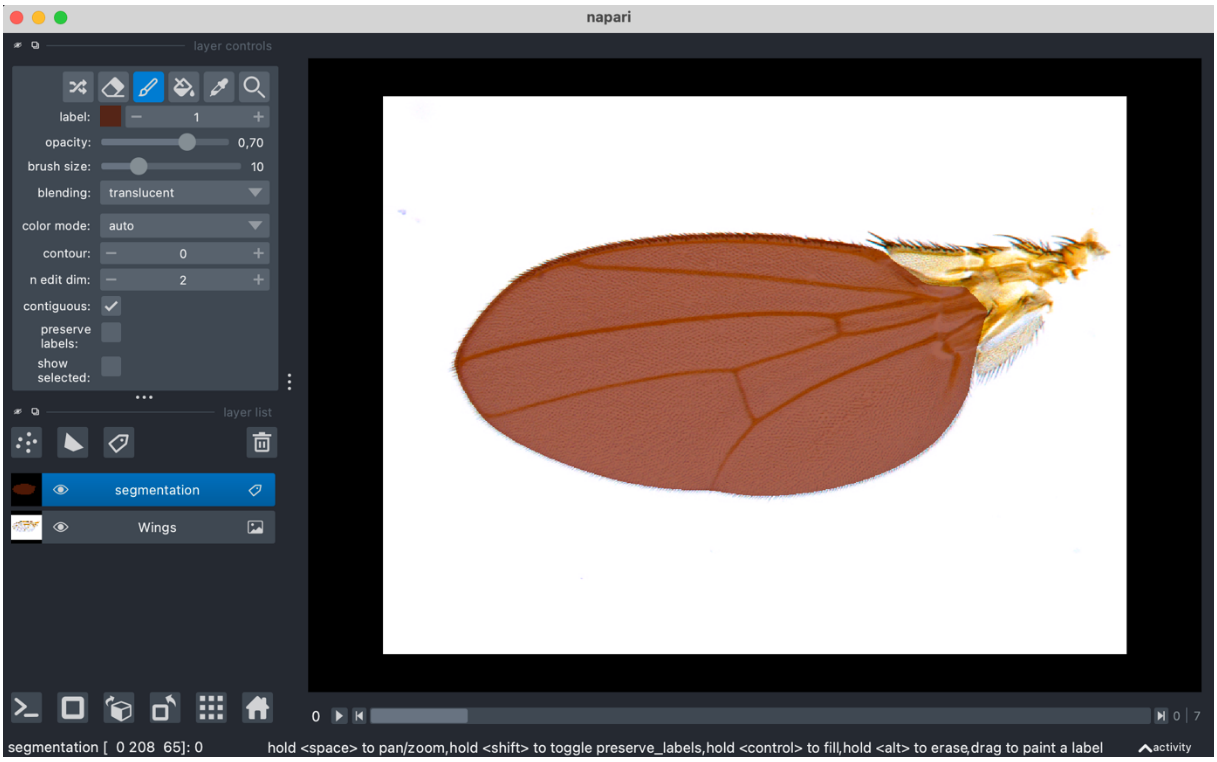

In this example, there is no segmentation mistake. Closing the Napari windows will automatically overwrite the results and masks folders, but here the area and asymmetry values will remain unchanged.
