# Supplementary figures and images for: A Dilp8-dependent time window ensures tissue size adjustment in Drosophila
Source: Nat Commun. 2022 Sep 26;13:5629. doi: 10.1038/s41467-022-33387-6 (PMC9512784; doi:10.1038/s41467-022-33387-6)

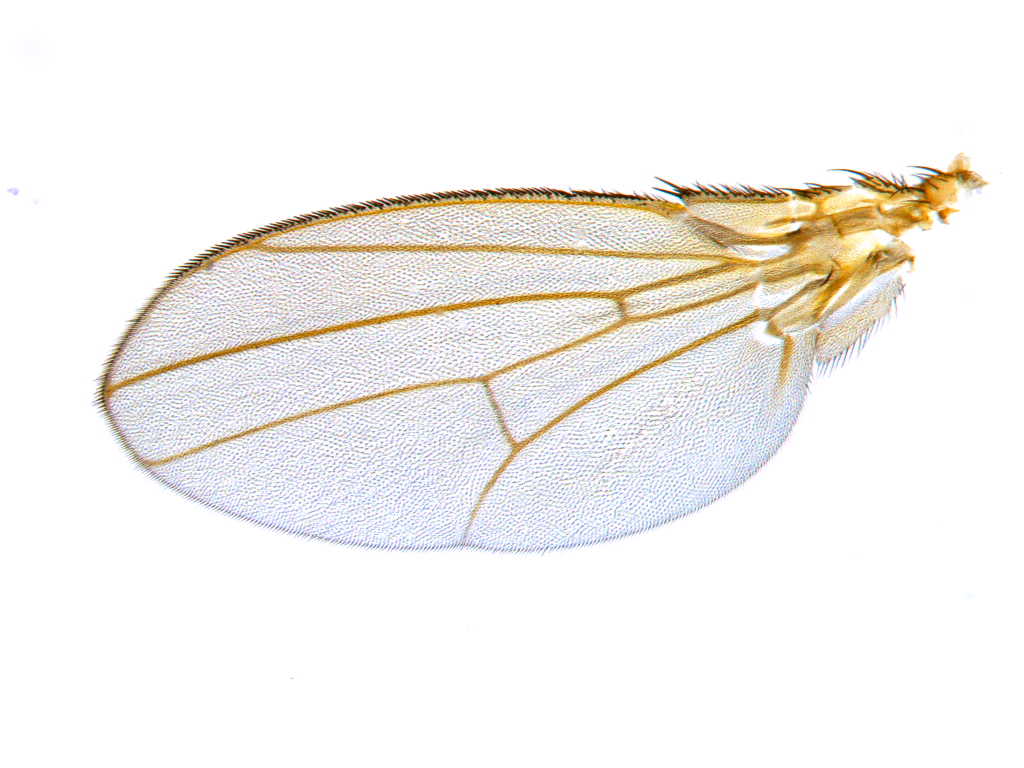

Supplement: Supplementary file 4 — Supplementary Data 1 [file 41467_2022_33387_MOESM4_ESM.zip › Supplementary Software Information/Demo/Wings pictures/D04L.tif]

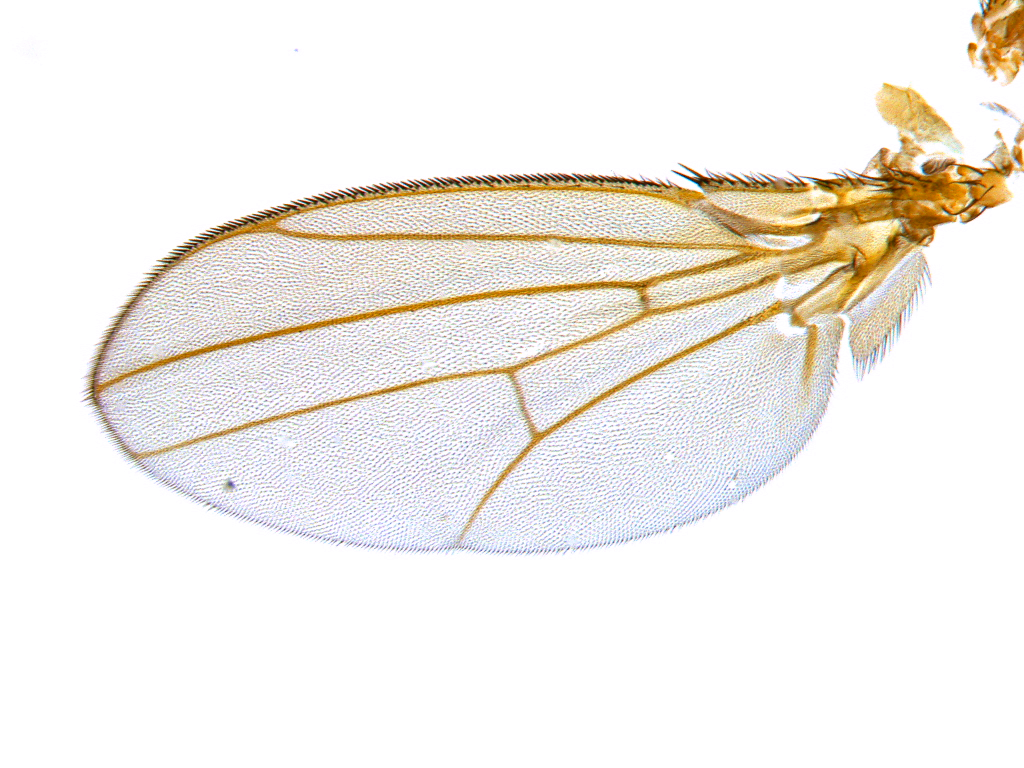

Supplement: Supplementary file 4 — Supplementary Data 1 [file 41467_2022_33387_MOESM4_ESM.zip › Supplementary Software Information/Demo/Wings pictures/D02L.tif]

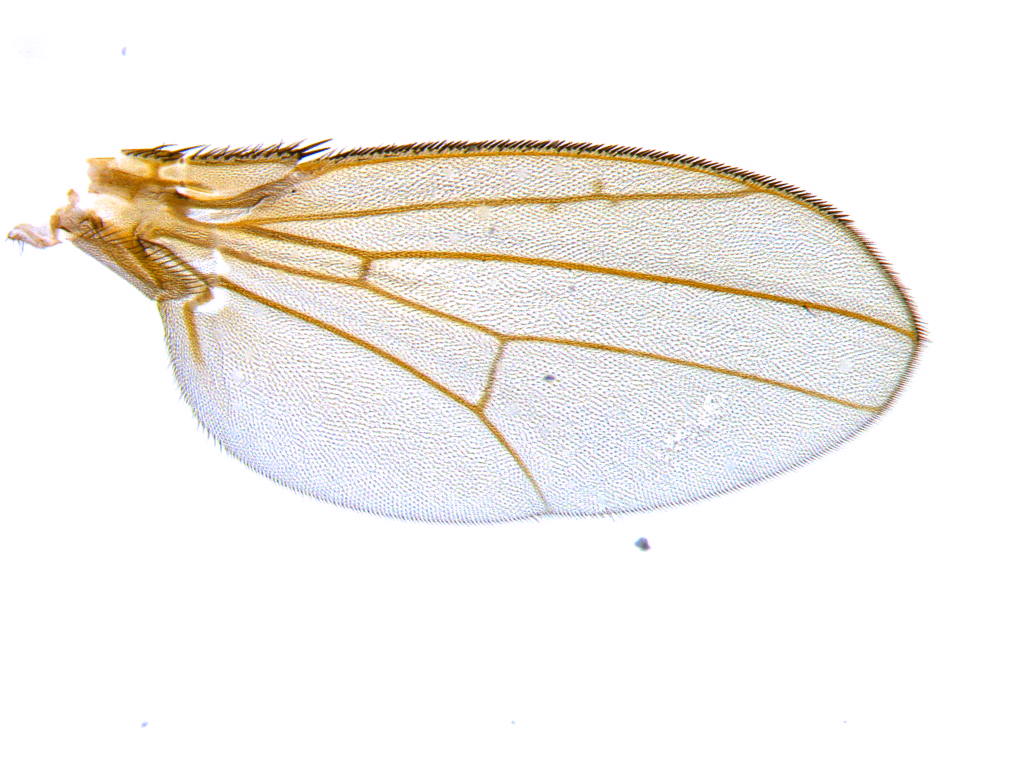

Supplement: Supplementary file 4 — Supplementary Data 1 [file 41467_2022_33387_MOESM4_ESM.zip › Supplementary Software Information/Demo/Wings pictures/D01R.tif]

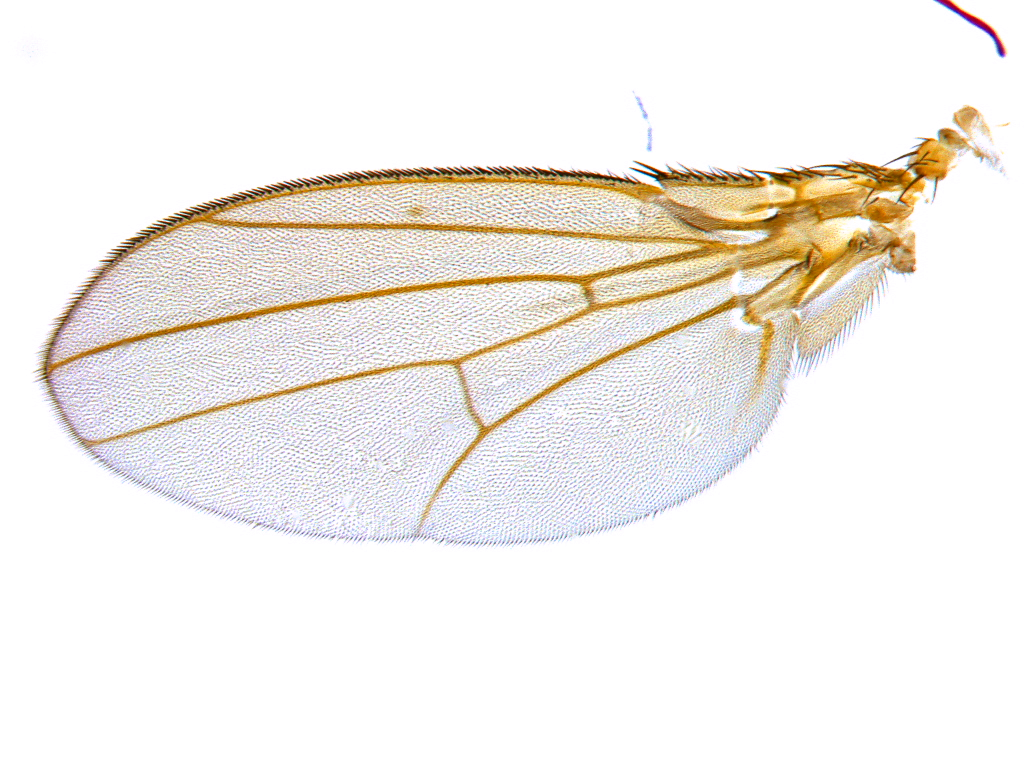

Supplement: Supplementary file 4 — Supplementary Data 1 [file 41467_2022_33387_MOESM4_ESM.zip › Supplementary Software Information/Demo/Wings pictures/D03L.tif]

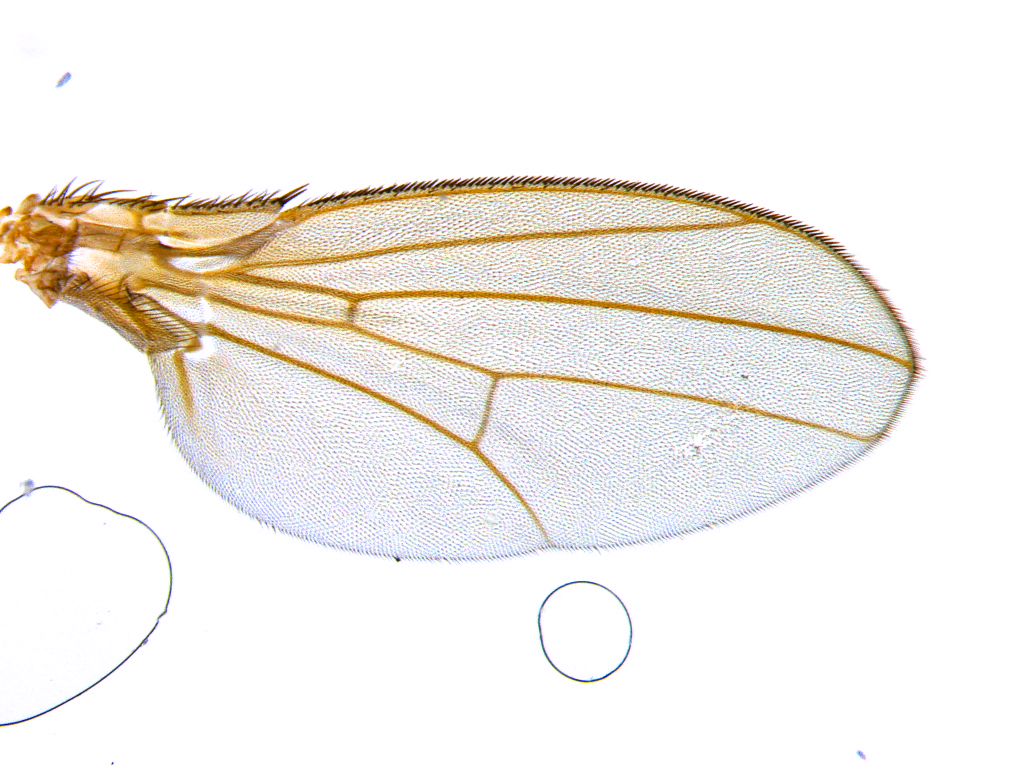

Supplement: Supplementary file 4 — Supplementary Data 1 [file 41467_2022_33387_MOESM4_ESM.zip › Supplementary Software Information/Demo/Wings pictures/D02R.tif]

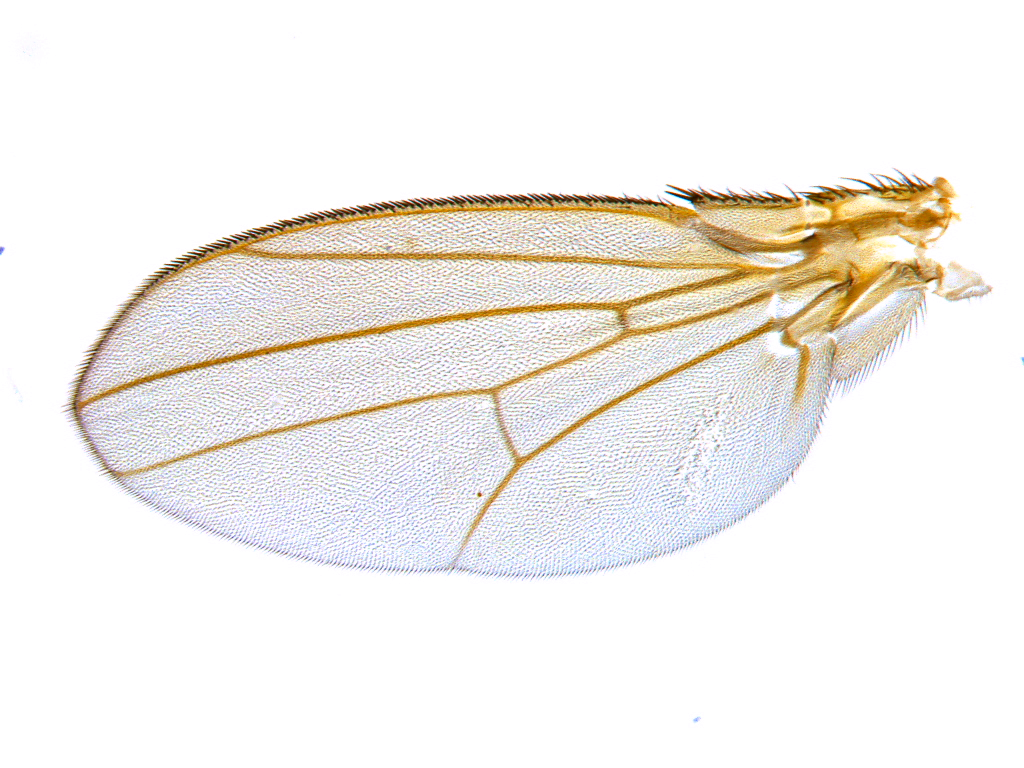

Supplement: Supplementary file 4 — Supplementary Data 1 [file 41467_2022_33387_MOESM4_ESM.zip › Supplementary Software Information/Demo/Wings pictures/D01L.tif]

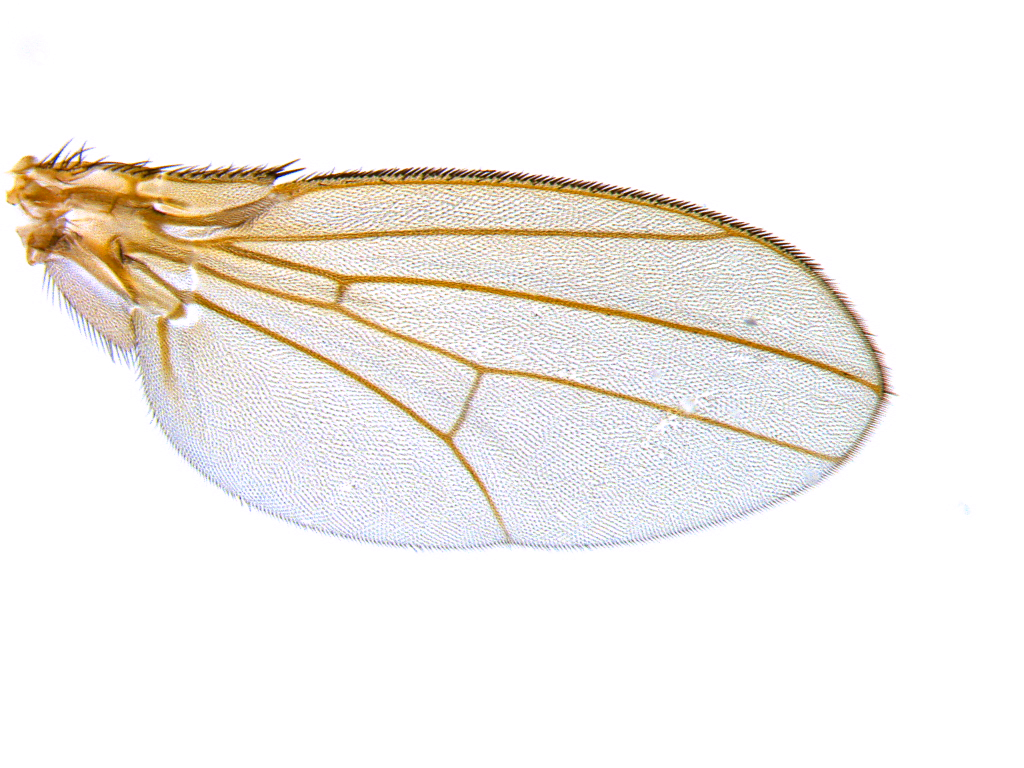

Supplement: Supplementary file 4 — Supplementary Data 1 [file 41467_2022_33387_MOESM4_ESM.zip › Supplementary Software Information/Demo/Wings pictures/D03R.tif]

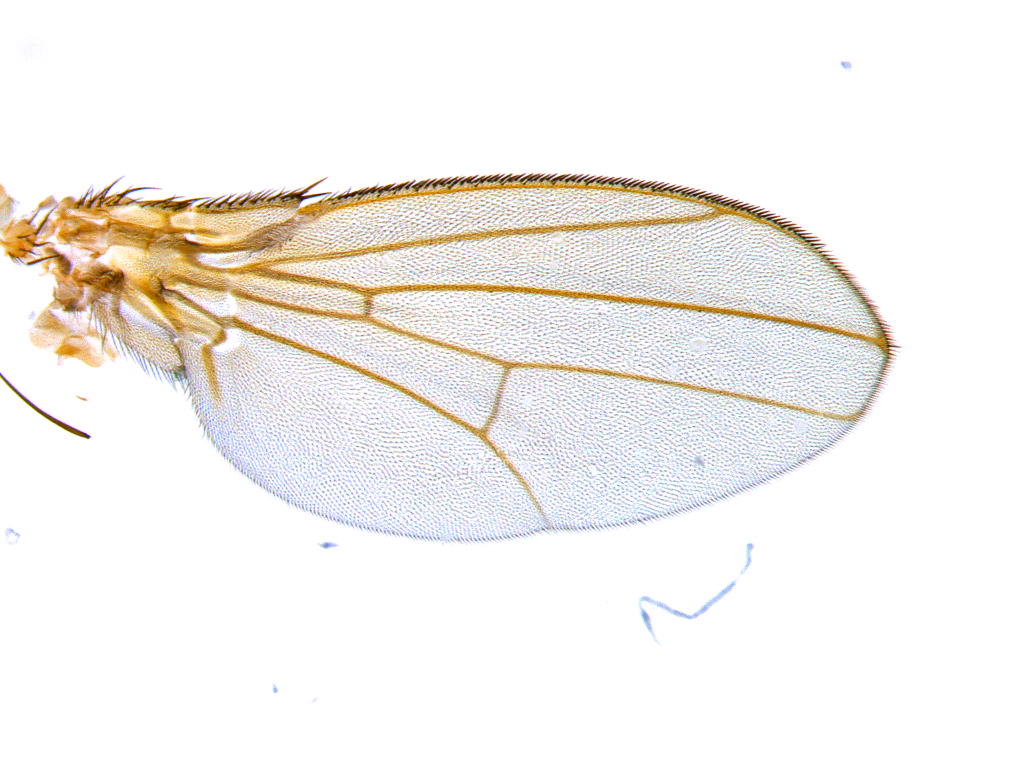

Supplement: Supplementary file 4 — Supplementary Data 1 [file 41467_2022_33387_MOESM4_ESM.zip › Supplementary Software Information/Demo/Wings pictures/D04R.tif]
