# Supplementary material for: A Dilp8-dependent time window ensures tissue size adjustment in Drosophila
Source: Nat Commun. 2022 Sep 26;13:5629. doi: 10.1038/s41467-022-33387-6 (PMC9512784; doi:10.1038/s41467-022-33387-6)
Supplement: Supplementary file 5 — Reporting Summary [file 41467_2022_33387_MOESM5_ESM.pdf]

nature portfolio

Corresponding author(s): Laura Boulan

Last updated by author(s): Aug 8, 2022

Reporting Summary

Nature Portfolio wishes to improve the reproducibility of the work that we publish. This form provides structure for consistency and transparency in reporting. For further information on Nature Portfolio policies, see our Editorial Policies and the Editorial Policy Checklist.

Statistics

For all statistical analyses, confirm that the following items are present in the figure legend, table legend, main text, or Methods section.

n/a	Confirmed	
☐	☒	The exact sample size (*n*) for each experimental group/condition, given as a discrete number and unit of measurement
☐	☒	A statement on whether measurements were taken from distinct samples or whether the same sample was measured repeatedly
☐	☒	The statistical test(s) used AND whether they are one- or two-sided<br>*Only common tests should be described solely by name; describe more complex techniques in the Methods section.*
☒	☐	A description of all covariates tested
☐	☒	A description of any assumptions or corrections, such as tests of normality and adjustment for multiple comparisons
☐	☒	A full description of the statistical parameters including central tendency (e.g. means) or other basic estimates (e.g. regression coefficient) AND variation (e.g. standard deviation) or associated estimates of uncertainty (e.g. confidence intervals)
☐	☒	For null hypothesis testing, the test statistic (e.g. *F*, *t*, *r*) with confidence intervals, effect sizes, degrees of freedom and *P* value noted<br>*Give P values as exact values whenever suitable.*
☒	☐	For Bayesian analysis, information on the choice of priors and Markov chain Monte Carlo settings
☒	☐	For hierarchical and complex designs, identification of the appropriate level for tests and full reporting of outcomes
☒	☐	Estimates of effect sizes (e.g. Cohen's *d*, Pearson's *r*), indicating how they were calculated

Our web collection on statistics for biologists contains articles on many of the points above.

Software and code

Policy information about availability of computer code

Data collection	Zeiss ZEN software for microscopy image acquisition (ZEN 3.2 blue edition).<br>LAS V4.12 for scope image acquisition.
Data analysis	Fiji open source image processing software (v1.53s)<br>Imaris software for 3D reconstructions (v8.4.2)<br>Microsoft Excel (v16.63.1) and GraphPad (v9.4.1) for statistical analysis

For manuscripts utilizing custom algorithms or software that are central to the research but not yet described in published literature, software must be made available to editors and reviewers. We strongly encourage code deposition in a community repository (e.g. GitHub). See the Nature Portfolio guidelines for submitting code & software for further information.

Data

Policy information about availability of data

All manuscripts must include a data availability statement. This statement should provide the following information, where applicable:

- Accession codes, unique identifiers, or web links for publicly available datasets
- A description of any restrictions on data availability
- For clinical datasets or third party data, please ensure that the statement adheres to our policy

Source data are provided with this paper.

Human research participants

Policy information about studies involving human research participants and Sex and Gender in Research.

Reporting on sex and gender	N/A
Population characteristics	N/A
Recruitment	N/A
Ethics oversight	N/A

Note that full information on the approval of the study protocol must also be provided in the manuscript.

Field-specific reporting

Please select the one below that is the best fit for your research. If you are not sure, read the appropriate sections before making your selection.

☒ Life sciences ☐ Behavioural & social sciences ☐ Ecological, evolutionary & environmental sciences

For a reference copy of the document with all sections, see nature.com/documents/nr-reporting-summary-flat.pdf

Life sciences study design

All studies must disclose on these points even when the disclosure is negative.

Sample size	No sample-size calculations were performed. The n-numbers were determined consistently with standard practices in Drosophila studies.
Data exclusions	No data were excluded in the analysis
Replication	All experiments were performed with at least three biologically independent samples. All qRT-PCR data presented was additionally obtained with three technical replicates for each sample. Technical replicates of wing area measurements for FA analysis were performed at least three times with no difference between each determination, due to the quantification automation using the custom-designed codes referred in the Code Availability section.
Randomization	Flies were allocated to experimental groups according to their genotype and developmental time.<br>The individuals inside each genotype were randomly chosen: Groups of 30 L1 larvae of the required genotype were randomly picked and put in vials containing fly food.<br>For microscopy image analysis, the regions and cells shown were randomly selected.
Blinding	Authors were blinded in all sample preparations for qRT-PCR experiments. For the rest of the experiments, blinding was not required because sample preparation, data collection and image analysis were performed using the same conditions for all the samples regardless of their identity.

Reporting for specific materials, systems and methods

We require information from authors about some types of materials, experimental systems and methods used in many studies. Here, indicate whether each material, system or method listed is relevant to your study. If you are not sure if a list item applies to your research, read the appropriate section before selecting a response.

Materials & experimental systems

n/a	Involved in the study	
☐	☒	Antibodies
☒	☐	Eukaryotic cell lines
☒	☐	Palaeontology and archaeology
☐	☒	Animals and other organisms
☒	☐	Clinical data
☒	☐	Dual use research of concern

Methods

n/a	Involved in the study	
☒	☐	ChIP-seq
☒	☐	Flow cytometry
☒	☐	MRI-based neuroimaging

Antibodies

Antibodies used

Chicken anti-GFP (Abcam, Ref # ab13970, Lot # GR236651-12).
Mouse anti-FasIII (Developmental Studies Hybridoma Bank, Ref # 7G10).
Mouse anti-beta-galactosidase (Promega, Ref # Z3781, Lot # 18637303).
Mouse anti-PH3, 1/200 (Cell Signaling, Ref # 9706S).
Goat anti-Mouse IgG (H+L) Highly Cross-Adsorbed Secondary Antibody, Alexa Fluor™ 546 ((Invitrogen, # A-11030, Lot # 2026145).
Goat anti-Mouse IgG (H+L) Highly Cross-Adsorbed Secondary Antibody, Alexa Fluor™ Plus 555 ((Invitrogen, # A32727, Lot # TE266003).
Goat anti-Mouse IgG (H+L) Highly Cross-Adsorbed Secondary Antibody, Alexa Fluor™ Plus 647 ((Invitrogen, # A32728, Lot # WE322197).
Goat anti-Chicken IgY (H+L) Secondary Antibody, Alexa Fluor™ 488 ((Invitrogen, # A-11039, Lot # 2304258).

Validation

Chicken anti-GFP (Abcam, Ref # ab13970), validated by IF on GFP-fusion proteins in Drosophila larval brains (Meschi et al., 2019, Developmental Cell 48, 76–86; https://doi.org/10.1016/j.devcel.2018.11.029). Other 2683 references cited by the manufacturer website can be found at: https://www.abcam.com/gfp-antibody-ab13970.html.

Mouse anti-FasIII (Developmental Studies Hybridoma Bank, Ref # 7G10). Validated in the references cited in The Antibody Registry: https://antibodyregistry.org/search.php?q=AB_528238

Mouse anti-beta-galactosidase (Promega, Ref # Z3781). Validated by references cited in the manufacturer website at: https://france.promega.com/resources/tools/citations/?p=3F2126E7-B5B1-47EE-9CB1-80F10DAF9107

Mouse anti-PH3, 1/200 (Cell Signaling, Ref # 9706S). Validated by references cited in the manufacturer website at: https://www.cellsignal.com/products/primary-antibodies/phospho-histone-h3-ser10-6g3-mouse-mab/9706.

Animals and other research organisms

Policy information about studies involving animals; ARRIVE guidelines recommended for reporting animal research, and Sex and Gender in Research

Laboratory animals

Animals of the Drosophila melanogaster species were used. The transgenic strains had w1118; yellow, w1118; or yellow, sc, v, sev backgrounds depending on the particular stocked used (information available within the Blomingtoon or Vienna stock collections with the stock reference number specified in the Methods section, "Fly strains" sub-heading). Also the individuals analyzed could have a heteroallelic combination of these backgrounds as a result of genetic crosses required for specific manipulations (Gal4 driver crossed to RNAi strain, for example). The age of the individuals used is specified in both the figures and figure legends in each experiment shown.

Wild animals

The study did not involve wild animals

Reporting on sex

Only female flies were used for FA studies. For the rest analysis, both females and males were used.

Field-collected samples

The study did not involve samples collected from the field.

Ethics oversight

No ethical approval was required. The study involves working with transgenic invertebrate laboratory animals (Drosophila melanogaster) and underlies the regulations of the safety level 1 containment in order to prevent spreading of transgenic animals in the environment. The study meets the EU and national legal and ethics requirements (EU Directive 2010/63/EU).

Note that full information on the approval of the study protocol must also be provided in the manuscript.
